# Principal Component Analysis and Molecular Characterization of Reniform Nematode Populations in Alabama

**DOI:** 10.5423/PPJ.OA.09.2015.0194

**Published:** 2016-04-01

**Authors:** Seloame T. Nyaku, Ramesh V. Kantety, Ernst Cebert, Kathy S. Lawrence, Joseph O. Honger, Govind C. Sharma

**Affiliations:** 1 Department of Crop Science, College of Basic and Applied Sciences, University of Ghana, P.O. Box LG 44, Legon-Accra; 2 Department of Natural Resources and Environmental Sciences, Alabama A & M University, Normal-AL, 35762; 3 Department of Entomology and Plant Pathology, Auburn University, Auburn, AL 36849; 4 Soil and Irrigation Research Centre, University of Ghana, P.O. Box LG 44, Legon-Accra

**Keywords:** principal component analysis, reniform nematode, variation

## Abstract

U.S. cotton production is suffering from the yield loss caused by the reniform nematode (RN), *Rotylenchulus reniformis*. Management of this devastating pest is of utmost importance because, no upland cotton cultivar exhibits adequate resistance to RN. Nine populations of RN from distinct regions in Alabama and one population from Mississippi were studied and thirteen morphometric features were measured on 20 male and 20 female nematodes from each population. Highly correlated variables (positive) in female and male RN morphometric parameters were observed for body length (L) and distance of vulva from the lip region (V) (r = 0.7) and tail length (TL) and c′ (r = 0.8), respectively. The first and second principal components for the female and male populations showed distinct clustering into three groups. These results show pattern of sub-groups within the RN populations in Alabama. A one-way ANOVA on female and male RN populations showed significant differences (*p* ≤ 0.05) among the variables. Multiple sequence alignment (MSA) of 18S rRNA sequences (421) showed lengths of 653 bp. Sites within the aligned sequences were conserved (53%), parsimony-informative (17%), singletons (28%), and indels (2%), respectively. Neighbor-Joining analysis showed intra and inter-nematodal variations within the populations as clone sequences from different nematodes irrespective of the sex of nematode isolate clustered together. Morphologically, the three groups (I, II and III) could not be distinctly associated with the molecular data from the 18S rRNA sequences. The three groups may be identified as being non-geographically contiguous.

The reniform nematode (RN) is distributed in most tropical and subtropical regions of the world, and within southern parts of U.S. and has a wide host range that includes vegetable and field crops such as economically important cotton (Robinson et al., 1997). Within the U.S., areas notably affected are Alabama (Gazaway et al., 2001), Louisiana (Overstreet, 1999), and Mississippi (Lawrence and McLean, 1999). The RN spread from one field to another is spurred by transfer of soil by farm equipment and vehicles (Carter et al., 2002). Under severe drought stress 50% of cotton yield losses can be experienced, but even under such stress conditions nematodes can survive and cause economic damage (Kirkpatrick and Robbins, 1998).

Cotton production in the U.S. occurs in approximately 30,000 farms which is approximately 13.5 million acres of land. The importance of cotton use world-wide cannot be over ruled, as consumers rely on its fiber, oil, and protein. However, upland cotton production is greatly limited by its susceptibility to the RN (Robinson, 1999), and this necessitates the need for novel control strategies for this pest. Traditionally, plant-parasitic nematodes have been managed through crop rotation, application of nematicides, and host resistance. Nematicidal use is not a desirable choice because of health, environment, and cost concerns. Host resistance, through the development of commercial upland cotton cultivars having resistance to the RN is therefore necessary (Cook and Evans, 1987; Koenning et al., 2004). Recently, there have been attempts to introgress RN resistance from *Gossypium longicalyx* through back-crossing into *G. hirsutum* (Robinson et al., 2007).

The 18S rRNA gene is known to be a slow evolving region in living organisms, however, it can be used for phylogenetic analysis for comparisons among taxa (Fitch et al., 1995; Nadler and Hudspeth, 1998). The 18S rRNA gene although is made up of several copies, has variation occuring within it, and this is often related to concerted evolution within the rDNA locus from crossovers and gene conversion events (Eickbush and Eickbush, 2007; Hillis and Dixon, 1991).

Intra-individual variation rarely occurs in the 18S rRNA gene of animal genomes. However, 18S rDNA variation has been observed in *Dugesia* (*Schmidtea*) *mediterranea*, a free-living platyhelminth (Carranza et al., 1996), *Meloidogyne* spp. (Huang et al., 2013; Powers et al., 2005), and in the RN (Nyaku et al., 2013; Tilahun et al., 2008). Within the RN 18S rRNA gene, two variants (RN_VAR1 and RN_VAR2) co-exist, with a 5% base difference among them.

Therefore understanding the genetic variability, structure, and shifts within RN populations, in relation to their morphology, dynamics, and behavior is needed to fully assess success of host plant resistance. Identification of variability in RN populations have been through morphometric characterization of populations (Agudelo et al., 2005; Nakasono, 1983; Nyaku et al., 2013; Soares et al., 2003). Agudelo et al. (2005) conducted a morphometric study using multivariate analysis of variance (MANOVA) and discriminant analysis on RN males and immature female populations. This showed considerable variation among the populations.

The objectives of this study were to utilize both morphological and molecular characterization of nematode populations to i) Locate the most useful morphometric characters in discriminating among ten RN populations using principal component analysis (PCA); ii) Determine within-nematode variation for three populations using the nuclear 18S rDNA regions in single female and male RN isolates from Alabama.

## Materials and Methods

### Soil Sample Collection and Establishment of Nematode Populations

**Fig. 1:**
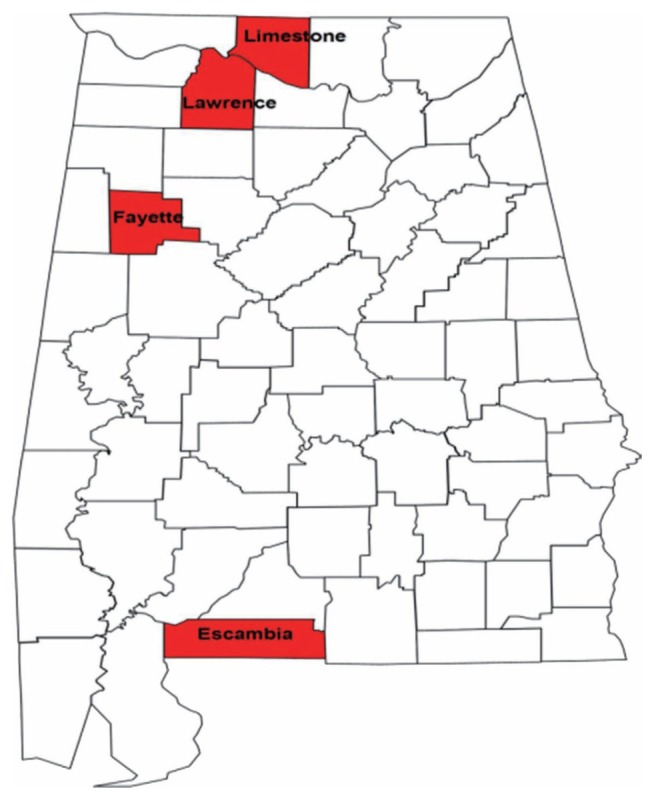
Reniform nematode collection sites in Alabama sampled for morphometric and molecular analysis.

Samples of RN-infested soil were obtained from ten farms (Table 1) located in four counties in Alabama (Fig. 1) and one location in Mississippi known to be infested with RN. Twenty soil cores, 2.5 cm in diameter and 20 cm in depth were collected from each farm in a systematic zigzag sampling pattern. The soil was then mixed thoroughly and 150 cm^3^ sub-sample was used for the extraction of the nematodes. The nematodes were extracted from the soil samples as described by Deng et al. (2008).

### Morphometric Measurements

These measurements were conducted on 20 individual female and male nematodes at the fourth juvenile (J4) stage from each field sample using an Olympus IMT-2 microscope (Olympus Optical Co. Ltd, Japan). Ten morphometric variables such as, body length (L), stylet length (SL), position of vulva (female nematodes) (V), spicule length (male nematodes) (SP), length of hyaline portion of tail (TL), position of dorsal esophageal gland orifice (DEGO), position of excretory pore (EP), maximum width (MW), esophageal length (ESOP) and anal width (AW), were measured. In addition, the de Man’s formula ratios *a* = body length/maximum body width, *b* = body length/esophageal length, *c* = body length/tail length, and, *c*′ = tail length/anal body width were calculated as described in earlier studies (Dasgupta et al., 1968).

### Statistical Analysis

Analysis of variance (ANOVA) and coefficient of variation (CV) were performed using Statistical Analysis System (SAS), version 9.2; SAS Institute Inc., Cary, NC, USA), on twelve variables (L, SL, TL, DEGO, EP, MW, ESOP, AW, *a*, *b*, *c*, and *c*′). The Tukey Studentized Range (HSD) test was used in determining significant differences *P* < 0.05 among the means for male and female measurements. Biplots were generated using Microsoft^®^ Excel 2000/XLSTAT^©^-Pro (Version 7.2*,* 2003, Addinsoft, Inc., Brooklyn, NY, USA); the significance level was set at *P* < 0.05 to determine the patterns of variation within individual female and male RN populations.

### DNA extractions from single female and male reniform nematodes

Soil samples infested with RN were obtained from three locations in Alabama: BelleMina (BM) in Limestone County (Group I), Lamons in Lawerence County (Group II), and Hamilton (HAM) in Lawerence County (Group III). These regions were chosen based on principal component analysis (PCA) of thirteen morphometric features performed on 9 RN-infested populations sampled in Alabama and one in Mississippi. Male and female RN were extracted from the soil samples according to Deng et al. (2008). DNA was then isolated from 10 female and 10 male nematodes using a DNeasy blood and tissue kit (Qiagen, Inc., Valencia, CA, USA) according to the manufacturer’s protocol.

### Polymerase Chain Reaction (PCR)

Two micro-liters of extracted DNA (~1.0 ng/μl) from a single RN was transferred into PCR tubes containing 2.5 μl 10× High Fidelity PCR buffer, 1.0 μl MgCl_2_ (50 mM), 0.5 μl dNTPs (10 mM), 0.5 μl of forward and reverse primers each (10 μm) (synthesized by MWG-Biotech AG, USA), 0.2 μl of high fidelity platinum taq (Invitrogen, Carlsbad, CA, USA) and sterile DNase free water added to a final volume of 25 μl. Primer pairs Nem_18S_F (5′-GGCGATCAGATACCGCCCTAGTT-3′) and Nem_18S_R (5′-TACAAAGGGCAGGGACGTATT-3′) were used in amplifying a 600 bp region of the 18S rRNA gene of the RN. Polymerase Chain Reaction (PCR) was performed in a Peltier Thermal Cycler (PTC) tetrad 2 DNA engine (Bio-Rad, Hercules, CA, USA). The PCR conditions were as follows: 94°C for 2 min, then 30 cycles of: 94°C for 30 sec, 60°C for 30 sec, and 68°C for 1 min. A final extension phase of 72°C for 7 mins concluded the amplification. The quality of PCR products was checked by electrophoresis of 6 μl of PCR product in 1% agarose gel with ethidium bromide staining. The bands were visualized and photographed under ultraviolet light. The size of each PCR product was then determined by comparison with a 100 bp DNA molecular marker.

### Cloning of PCR Products

Polymerase Chain Reaction (PCR) products from 10 individual female and 10 male nematodes was purified before cloning using a QIAquick PCR Purification Kit (Qiagen, Inc., Valencia, CA, USA) according to the manufactures protocol. The purified fragments were then cloned into a plasmid vector using TOPO TA Cloning Kit (Invitrogen, Carlsbad, CA, USA). The ligation reaction was made up of 4 μl of PCR product, 1 μl of salt solution (1.2 M NaCl and 0.06 M MgCl_2_), and 1 μl of TOPO vector. Several clones were picked for verification of inserts from PCR amplifications for each selected clone through colony PCR. This was performed by amplification of the clonal DNA using M13 forward (5′-TGTAAAACGACGGCCAGT-3′) and reverse (5′-AGCGGATAACAATTTCACAC-3′) primers. PCR conditions were as follows: 94°C for 5 min, then 40 cycles of the following: 94°C for 30 sec, 55°C for 1 min, and 72°C for 1 min. The final extension phase was 72°C for 10 min. Individual bacterial colonies with inserts were picked and placed into 96-well blocks with 1.3 ml of liquid Luria- Bertani (LB) media containing 100 μg/ml ampicillin and shaken at 37°C for 24 hours at 300 rpm in an Innova 4300 rotary incubator shaker (New Brunswick Scientific, Edison, NJ, USA). The bacterial cells in 96 well blocks were then centrifuged for 12 min at 2,000 × g in an Eppendorf 5804R centrifuge (Brinkmann Instruments Inc., Westbury, NY, USA) to obtain cell pellets. Plasmid DNA from the bacterial cells was isolated using a QIAprep Mini-prep kit (Qiagen, Inc., Valencia, CA, USA) according to the manufacturer’s protocol.

### Sequencing

The 18S rRNA genes of the RN were sequenced using T7 (5′-TAATACGACTCACTATAGGG-3′) and T3 (5′-TAACCCTCACTAAAGGGA-3′) primers. Plasmid inserts from at least ten clones originating from each of the ten nematodes were sequenced using the ABI PRISM Big Dye Terminator cycle sequencing ready reaction kit (Applied Biosystems, Foster City, CA, USA) in an ABI 3730 nucleotide sequencer and screened for homology to nematoda sequences using the standard nucleotide-nucleotide BLAST [blastn] on the NCBI website (http://www.ncbi.nlm.nih.gov/Blast.cgi). The 18S rRNA sequences from the Hamilton and Lamons populations have been deposited into GenBank under the accession numbers KR152657-KR152823 and KR152824-KR153037 respectively.

### Alignment and Phylogenetic Analysis

This analysis was initiated with inclusion of 18S rRNA sequences from five other RN populations, together with sequences from the current study. These 18S rRNA clone sequences were from Belle Mina female (12) and male RN population (12) (Nyaku et al., 2013), Mixed-Alabama populations (12) (Nyaku et al., 2013), and other Alabama populations (4) (Tilahun et al., 2008) (Table 2).

The SeqMan Pro program within the DNASTAR Lasergene v8.0 software (DNASTAR Inc., Madison, WI, USA) was used in generating consensus sequences from both forward and reverse sequences and any extraneous sequences outside the respective amplification fragments trimmed-off. Multiple sequence alignment and phylogenetic analysis were conducted using Molecular Evolutionary Genetics Analysis (MEGA) software version 6.0 (Tamura et al., 2013).

## Results

### Eigenvalues

Thirteen eigenvalues were noted for both female and male RN populations for the measured morphometric parameters. High variability was observed for the components (PC1 and PC2) compared to the other components. The cumulative variability’s for PC1 and PC2 were 45.0% and 49.5% for female and male populations, respectively.

### Eigenvectors

The eigenvectors contribute to the coefficient of variables either negatively or positively. Majority of the eigenvectors contributed positively to PC1 and PC2 (Table 3). Both female and male RN populations had excretory pore and ‘c’ contributing negatively to PC1. However, tail length, maximum width, anal with length, and c′ contributed negatively to PC2 for both female and male RN populations.

### Pearson’s correlations among variables (V) for female and male populations

Variables were either positively or negatively correlated in both female and male RN populations (Table 4). Highly correlated variables (positive) in female and male RN morphometric parameters were between body length and distance of vulva from the lip region (r = 0.7) and tail and c′ (r = 0.8) respectively. Similarly, negative correlations were also observed among variables for both female and male RN populations. Within the female populations, c and tail length (r = −0.8); together with anal width and maximum body width (r = −0.7) were negatively correlated. The male populations however had c and tail length (r = −0.9); b and esophageal length (r = −0.7); and c′ and c (r = −0.8) negatively correlated.

### Correlations between variables and principal components

Variates were either positively or negatively correlated with the components in both female and male RN populations (Table 5). Highly correlated variables (positive) with PC1 were vulva (r = 0.71), and anal width (r = 0.79) for female populations. The male populations however, had tail length (r = 0.95), DEGO (r = 0.78), and c′ (r = 0.73) being highly correlated with PC1. Negatively correlated variables with PC1 were, c′ (r = −0.73) and c (r = −0.87) for female and male RN, respectively. Other sets of variates were highly correlated with PC2 for both female and male RN populations. Highly correlated variables (positive) with PC2 were a (r = 0.77) and c (r = 0.73) for female populations. The male populations however, had only a (r = 0.90) being highly correlated with PC 2. Negatively correlated variables with PC 2 were however below r = −0.40 and r = −0.60 for female and male RN, respectively.

### Principal component analysis for female and male RN Populations

**Fig. 2:**
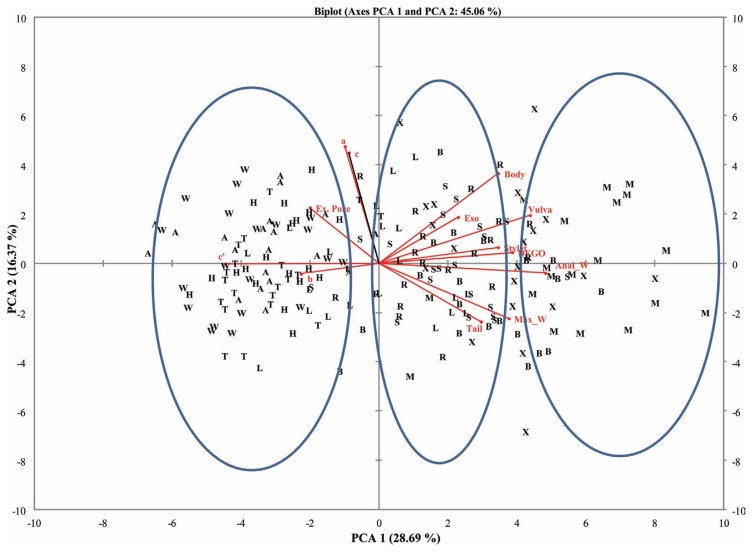
Biplot for 10 female reniform nematode populations from four counties for PCA 1 and 2.

**Fig. 3:**
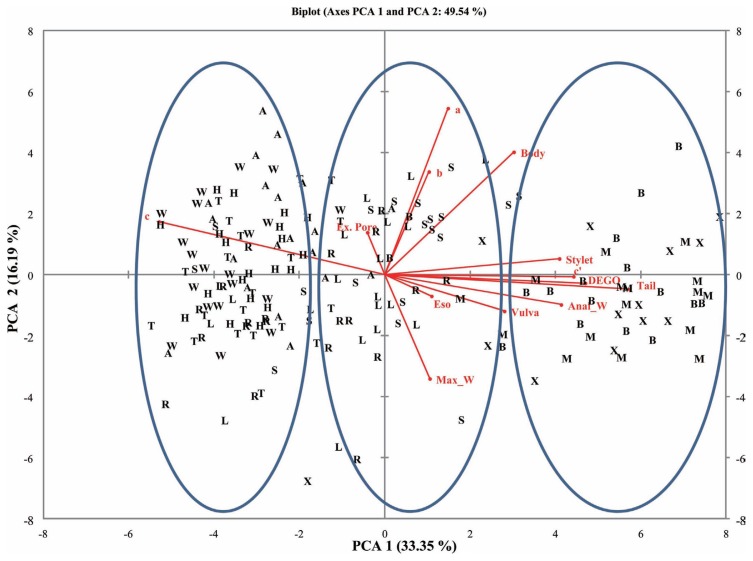
Biplot for 10 male reniform nematode populations from four counties for for PCA 1 and 2.

A plot of the first and second principal components (PC1 and PC2) for the female and male populations showed populations clustering into three groups. Populations from Belle Mina (Limestone County, AL), Huxford (Escambia County, AL), and MSU (Oktibbeha County, MS) (Group I) were more closely related compared to populations from Shaw (Limestone County, AL), Murphy (Limestone County, AL), and Lamons (Fayette County, AL) (Group II) and populations from Hargrave (Limestone County), Hamilton (Lawrence County), Thorton (Lawrence County), and Whitehead (Fayette County) (Group III) (Figs. 2 and 3). Biplots for female populations indicate, body length, stylet length, position of vulva, DEGO, anal width, maximum width, and length of hyaline portion of tail serving as variables distinguishing the group 1 populations from the other populations. Esophageal length, ‘c’, and ‘a’ separated the group two populations. The position of excretory pore, c′, and b separated the group III populations. Variables in the biplots for the male RN populations were varied from those that separated the female populations. The group I male populations clustered based on stylet length, DEGO, tail length, anal width and c′. Bodylength, position of vulva, esophageal length, maximum width, ‘a’, and ‘b’, separated the group II populations. However, a single variable ‘c’ separated group III populations.

### Analysis of Variance (ANOVA)

Analysis of variance separately on female and male populations showed significant differences (*p* ≤ 0.05) in all the measurements for the female populations with the exception of *a* and *c*. The male populations also had significant differences (*p* ≤ 0.05) in all the measurements (Data not shown). A one-way ANOVA on the combined female and male RN populations also showed significant differences (*p* ≤ 0.05) among the variables (Table 6). In relation to body length (L), only the population from WhiteHead showed significant differences between female and male populations. The population from Huxford had no significant differences between female and male populations for stylet length (SL), however, SL was significantly different between female and male populations from other locations. There were also significant differences among female and male RN populations for the variables *a*, *b*, *c*, and *c*′ with some exceptions. Populations without any significant differences between female and male populations were Murphy, BelleMina and Murphy, Shaw and Lamons, and Hamilton and WhiteHead for the variables *a*, *b*, *c*, and *c*′ respectively.

### Coefficient of Variation

Coefficient of variation (CV) values for the various variables ranged from 0.0 to 28.8 (Table 7). Variables with the least ranges (6.5–8.1) were observed on L for Hargrave and Huxford populations respectively. The largest range (7.1–28.2) for the variables were noted on ESOP for Thorton and MSU respectively. The population from Hamilton had the lowest (0.0) CV for AW. Stylet length ranged from 6.0 to 20.2 with the lowest variation observed in Thorton and the highest variation observed in Huxford. Tail length ranged from 8.1 to 16.6 with the lowest variation observed in Thorton and the highest variation observed in Mississippi. Variation in CV for the de Man ratios were clearly observed. These ranged from 7.3 to 20.8 on *a* and *c*′ for Hargrave and BelleMina populations respectively.

### Mean values for male and female reniform nematode populations

The mean and standard deviation (SD) values from eight variables for male and female RNs populations revealed significant variability’s among them (Table 8). Body length measurements for males ranged from 270 μm to 440 μm. There were no significant differences (*p* ≤ 0.05) for L in the male populations, however, BelleMina populations had the highest mean value of 390.5 μm. All other variables had significant differences (*p* ≤ 0.05) among them. Stylet length ranged from 11.9 μm to 21.4 μm with the population from Huxford having the highest mean value of 14.9 μm. The highest SD values were observed in L and these ranged from 20.3 to 32.7 for male populations from Hargrave and Huxford respectively. The male population from Huxford had the highest SD value of 2.7 for SL. This population also had the greatest variation in SL for all the male populations. The mean of SL in male Huxford populations was significantly different (*p* ≤ 0.05) from Hargrave and Whitehead populations. Body length measurements for the female populations ranged from 300 μm to 450 μm. The population from Mississippi had the highest mean value of 386.5 μm for body length. Female Mississippi populations had significantly different (*p* ≤ 0.05) mean values from Thorton and Whitehead populations. Hargrave, Thorton, WhiteHead, male populations and Thorton female population had SD values of 0.0 from the mean for SL. The SD values for the other populations fell within the range of 0.8 and 1.9 for this variable. The means of tail length for male populations from Mississippi, Huxford, and BelleMina were significantly different (*p* ≤ 0.05) from the male population from Whitehead. The means of tail length for female populations from BelleMina and Mississippi were significantly different (*p* ≤ 0.05) from the Whitehead population. The mean for maximum width for the male population from Mississippi was significantly different (*p* ≤ 0.05) from the mean of the population from Hamilton. The female populations, the mean for maximum width for the population from BelleMina was significantly different from the population from Hamilton. The mean values for anal width for the male populations from BelleMina and Huxford were significantly different from populations from Hargrave, Hamilton, Thorton, and WhiteHead. The female populations from Huxford, Mississippi, and BelleMina were significantly different from the populations from populations from Thorton, Whitehead, Hargrave, and Hamilton.

### Sequencing Analysis for 18S rRNA gene

The 18S rRNA sequences comprised of 73, 94, 104, and 110 sequences from Hamilton female and male, and Lamons female and male populations respectively. Additional 18S rRNA clone sequences were from BelleMina female (12) and male RN population (12) (Nyaku et al., 2013), Mixed-Alabama populations (12) (Nyaku et al., 2013), and other Alabama populations (4) (Tilahun et al., 2008).

**Fig. 4:**
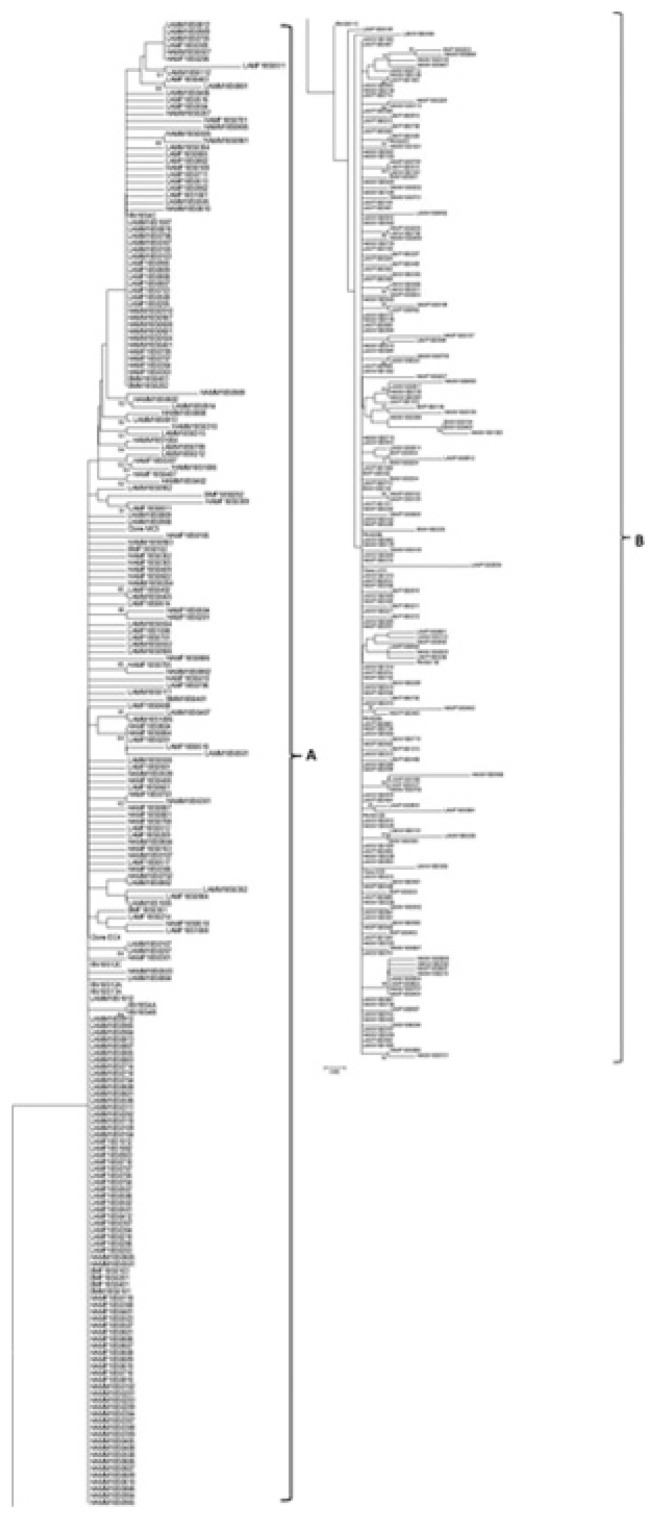
Phylogram generated from Neighbor-Joining analysis for 18S rRNA clone sequences of female and male reniform nematodes from mixed-Alabama, Belle Mina, Hamilton, and Lamons populations. The percentages of bootstrap replicates supporting the clades are indicated at the branch points. Bootstrap values greater than 50% are shown beside nodes. RN = Reniform nematode; 18S = 18S rRNA gene; 01–10 = reniform nematode isolate IDs; A, B, C/01–16 = clone IDs, BM = Belle Mina, LAM = Lamons, HAM = Hamilton, F = Female, M = Male.

Multiple sequence alignment (MSA) of 18S rRNA sequences (421) showed lengths of 653bp. Sites within the aligned sequences were conserved (53%), parsimony-informative (17%), and singletons (28%), indels (2%) respectively. These regions showed no base changes, two or more base changes, single base changes, and insertion and deletions respectively. Neighbor-Joining analysis showed two major groupings (A and B) (Figs. 4A and 4B, respectively) for the various clone in all the populations. Within each of these groups A and B, clone sequences from different nematodes irrespective of the sex of nematode isolate clustered together. The clone sequence MC5 and EC4 clustered in group A. The clone MC5 clustered on the same branch as clone sequences (LAMM18S0809 and LAMM18S0908). The clone sequence EC4 clustered on the same branch close to LAMM18S0107 and LAMM18S0207. The clone sequences MC6 and LH10 clustered in group B. The sequence from MC6 was similar to LAMM18S0210, and LH10 clustered close to LAMM18S1010, LAMF18S0515, and HAMF18S0508, all clones were from RN obtained from Lawrence County. Twenty-seven parsimony informative sites were used in distinguishing the two major groups (Supplementary Table 1).

### Evolutionary relationships of taxa

The evolutionary history was inferred using the Neighbor-Joining method (Saitou and Nei, 1987). The percentage of replicate trees in which the associated taxa clustered together in the bootstrap test (1,000 replicates) is shown next to the branches (Felsenstein, 1985). The tree is drawn to scale, with branch lengths in the same units as those of the evolutionary distances used to infer the phylogenetic tree. The evolutionary distances were computed using the Maximum Composite Likelihood method (Tamura et al., 2004) and are in the units of the number of base substitutions per site. The analysis involved 421 nucleotide sequences. All positions containing gaps and missing data were eliminated. There were a total of 382 positions in the final dataset. Evolutionary analyses were conducted in MEGA6 (Tamura et al., 2013).

## Discussion

Principal Component Analysis (PCA) was used in grouping and distinguishing among ten populations of RN. This analysis is a multivariate technique for examining relationships among several quantitative variables, and allows for the detection of linear relationships. Morphometric variability among RN populations has been reported by several authors (Dasgupta et al., 1968; Linford and Oliveira, 1940; Nakasono, 1983; Robbins, 1994; Sivakumar and Seshadri, 1971; Soares et al., 2003). Correlations among the variables were either positive or negative. Highly correlated variables (positive) in RN morphometric parameters were between body length and distance of vulva from the lip region (0.7) and tail length and c′ (0.8) for female and male populations, respectively. The distance of vulva from the head region and anal width were highly correlated with PC1 in the female populations. This underscored the importance of vulval position in differentiating female populations between locations and Groups. But in male populations, tail length, DEGO, and c′ being highly correlated with PC1, an indication of their usefulness in distinguishing among male populations. The absence of significant differences among female and male RN populations for body length in 9 out of 10 populations made this variable less important in distinguishing among female and male RN populations. The Group I populations showed significant differences for both female and male populations in relation to anal width which will be a useful variable in discriminating among populations. Furthermore, ANOVA also showed significant differences among female and male populations for maximum width. This variable will also be useful for discriminating among RN populations.

Our study revealed both female and male populations from Huxford having the greatest variation in body lengths. Reports made previously of immature females ranged from 291 μm in the U.S (Robbins, 1994) to 514 μm in India (Sivakumar and Seshadri, 1971). There was an overlap in the body length measurements between female and male RN populations. Our findings are similar to those of Agudelo et al. (2005), with a high variability in morphometrics within populations in the southern United States. A comparison of measurements for body length of immature female RN made from Huxford location by Agudelo et al. (2005) were similar to those observed here in our study showed a similarity to the maximum value of 430 μm. However, the minimum body length values differed slightly with the value from our study being lower. The range of measurements made for male RN from Huxford by Agudelo et al. (2005) for body length was from 375 μm to 480 μm. This however, differed from our measurements which ranged from 270 μm to 420 μm. Longer body lengths of *R. reniformis* have been reported from Indian populations (Sivakumar and Seshadri, 1971).

Coefficient of variation (CV) revealed values that ranged from 5% to 15% (Table 7). Four of the populations, Hargrave, Thorton, Hamilton, and WhiteHead had all their variables with CV less than 12%. These populations were always grouped together for both female and male RN, an indication that all variables were useful in their grouping. A study by Ye and Robbins (2004), on *Longidorus* species had most of the variables measured with 5% to 15% CV. These variables were used in stepwise discriminant analysis to distinguish among the species. Populations from BelleMina, Huxford, and Mississippi which had at least six of their variables with CV greater than 12% and at least one variable with CV greater than 19% (Table 7) were grouped together for both female and male populations. Likewise populations from Shaw, Murphy, and Lamons were also grouped together for both female and male populations. These populations had at least 3 variables with CV greater than 12%.

Principal component analysis revealed three distinct groups (I, II, III). A population from each of these three groupings [BelleMina (BM, Limestone County, Group I), and Lamons (LAM, Lawerence County, Group II), and Hamilton (HAM, Lawerence County, Group III)] was selected for sequencing using the 18S rRNA gene region with the aim of comparing both morphometric and molecular data in distinguishing among RN populations. Neighbor-joining analysis showed the presence of both intra- and inter-nematode variation within the 18S rRNA gene from single female and male RN isolates. This variation was evident in the clustering of clones from an individual nematode with those from other RN from varying Counties. This variation has been observed within the RN 18S rRNA gene by other investigators (Nyaku et al., 2013; Tilahun et al., 2008). The presence of 27 parsimony-informative sites within both groups (A and B) for the RN populations is a confirmation of the presence of variants within the 18S rRNA gene. A previous study by Nyaku et al. (2013) revealed two variants types (RN_VAR1 and RN_VAR2) within the 18S RNA gene of the RN, with a 5% difference distinguishing among the variants. Variation within the 18S gene of *Meloidogyne* spp. has also been identified. However, this gene could not be used to efficiently distinguish among *M. arenaria*, *M. incognita*, and *M. javanica* (Huang et al., 2013; Powers et al., 2005). Variation within the 18S rRNA gene can be related to concerted evolution ongoing within the rDNA locus from crossovers and gene conversion events. Unequal crossover occurs between sister chromatids and this leads to variation in the rRNA gene (Eickbush and Eickbush, 2007).

This study with its focus on principal component analysis allowed us to examine both the linear relationships and the relative importance of thirteen quantitative variables measured and analyzed here. These findings provides new and additional information to the previous study by Nyaku et al. (2013) and underscore the relative importance of vulval position and anal width measurements to be of critical importance among female RN populations, and body length among male RN populations. The vulval position and body lengths for female and male RN from investigations by Nyaku et al. (2013), could be considered important variables for discriminating between populations because, their analysis revealed absolute values of 0.89 and 0.86 for the between canonical variate 1 (CAN1) structure for female and male reniform nematodes. Both of these values were significant at *p* ≤ 0.001. In this study, we have also incorporated new sets of 18S rRNA sequences from individual male and female RN for variation detection if present in populations. Morphologically, the three groups (I, II and III) characterized and the molecular data from the 18S rRNA sequences, revealed the presence of significant biological variation in the RN populations. The 18S rRNA showed inter and intra-nematodal variation in a single RN irrespective of sex and location. The numerous clone sequences obtained through sequencing the 18S rRNA gene from individual female and male RN in each of the three groups, clustered together irrespective of the nematode’s population or group.

The three groups may be non-geographically contiguous and heterogeneous. This study further infers that, significant morphological variation exist within female and male RN populations across Alabama, and the population from Mississippi was not distinct from populations sampled within Alabama.

## Supplementary Information



## Figures and Tables

**Table 1:** Nematode population sampling sites in Alabama & Mississippi

Farm Location	County/State	Abbreviation
BelleMina	Limestone, AL	B
Shaw	Limestone, AL	S
Murphy	Limestone, AL	R
Hargrave	Limestone, AL	H
Thorton	Lawerence, AL	T
Hamilton	Lawerence, AL	A
Lamons	Lawerence, AL	L
Whitehead	Fayette, AL	W
Huxford	Escambia, AL	X
MSU	Oktibbeha, MS	M

**Table 2:** List of clone sequences and their accession numbers obtained from GenBank

CLONE	ACCESSION #	CLONE	ACCESSION #	CLONE	ACCESSION #	CLONE	ACCESSION #
BMF18S0102	KF019952	BMM18S0101	KF020042	RN18S4A	JN695066	MC5	AY373545
BMF18S0103	KF019953	BMM18S0102	KF020043	RN18S4B	JN695067	EC4	AY373561
BMF18S0104	KF019954	BMM18S0104	KF020044	RN18S4C	JN695068	MC6	AY373543
BMF18S0201	KF019960	BMM18S0202	KF020052	RN18S5A	JN695073	LH10	AY373568
BMF18S0202	KF019961	BMM18S0203	KF020053	RN18S5B	JN695074		
BMF18S0203	KF019962	BMM18S0204	KF020054	RN18S5C	JN695075		
BMF18S0301	KF019971	BMM18S0301	KF020063	RN18S11A	JN695081		
BMF18S0302	KF019972	BMM18S0303	KF020064	RN18S11B	JN695082		
BMF18S0303	KF019973	BMM18S0304	KF020065	RN18S11C	JN695083		
BMF18S0401	KF019981	BMM18S0401	KF020073	RN18S12A	JN695084		
BMF18S0403	KF019982	BMM18S0403	KF020074	RN18S12B	JN695085		
BMF18S0404	KF019983	BMM18S0405	KF020075	RN18S12C	JN695086		

**Table 3:** Eigenvectors for female and male reniform populations

	Female		Male	
	
	PC1	PC2	PC1	PC2
L	0.292	0.408	0.240	0.457
SL	0.291	0.072	0.324	0.060
V	0.370	0.218	0.222	−0.136
TL	0.250	−0.265	0.455	−0.054
DEGO	0.326	0.050	0.373	−0.032
EP	−0.169	0.253	−0.032	0.157
MW	0.318	−0.250	0.084	−0.388
ESOP	0.194	0.209	0.087	−0.080
AW	0.407	−0.044	0.328	−0.112
a	−0.084	0.530	0.117	0.620
b	−0.191	−0.048	0.082	0.384
c	−0.074	0.502	−0.419	0.198
c′	−0.378	−0.003	0.351	−0.007

L = body length; SL = stylet length; V = Position of vulva; TL = tail length; DEGO = dorsal esophageal gland orifice; EP excretory pore; MW = maximum width; ESOP = Esophageal length; AW = Anal Width; *a* = body length/maximum body width; *b* = body length/esophageal length; *c* = body length/tail length *c*′ = tail length/anal body width

**Table 4:** Pearson Correlation among variables for female and male reniform nematode populations

Variables (Females)	L	SL	V	TL	DEGO	EP	MW	ESOP	AW	*a*	*b*	*c*	*c*′
L	1												
SL	0.181	1											
V	0.703	0.195	1										
TL	0.290	0.170	0.385	1									
DEGO	0.297	0.458	0.338	0.251	1								
EP	0.094	−0.179	−0.102	−0.190	−0.131	1							
MW	0.305	0.130	0.356	0.244	0.267	−0.113	1						
ESOP	0.249	0.478	0.187	0.071	0.327	−0.023	0.079	1					
AWW	0.306	0.328	0.485	0.266	0.360	−0.323	0.417	0.075	1				
*a*	0.430	−0.005	0.186	−0.021	−0.046	0.168	−0.718	0.080	−0.131	1			
*b*	−0.051	−0.064	−0.340	−0.070	−0.009	−0.014	−0.096	−0.191	−0.446	0.029	1		
*c*	0.278	−0.044	0.023	−0.822	−0.071	0.222	−0.075	0.077	−0.068	0.271	0.026	1	
*c*′	−0.284	−0.437	−0.343	−0.042	−0.412	0.289	−0.458	−0.106	−0.688	0.214	0.242	−0.137	1

Variables (Males)	L	SL	SP	TL	DEGO	EP	MW	ESOP	AW	*a*	*b*	*c*	*c*′

L	1												
SL	0.367	1											
V	0.115	0.200	1										
TL	0.376	0.499	0.382	1									
DEGO	0.307	0.564	0.291	0.636	1								
EP	0.133	−0.111	−0.010	−0.066	0.048	1							
MW	0.092	0.079	0.178	0.169	0.078	−0.014	1						
ESOP	0.184	0.140	0.025	0.107	0.275	0.081	−0.036	1					
AW	0.242	0.502	0.274	0.611	0.569	−0.144	0.248	0.008	1				
*a*	0.658	0.216	−0.046	0.158	0.175	0.105	−0.680	0.179	0.010	1			
*b*	0.524	0.113	0.058	0.148	−0.033	0.025	0.098	−0.734	0.146	0.291	1		
*c*	−0.110	−0.452	−0.373	−0.938	−0.604	0.133	−0.143	−0.091	−0.595	0.017	0.021	1	
*c*′	0.299	0.288	0.297	0.835	0.418	−0.018	0.056	0.136	0.091	0.176	0.076	−0.786	1

L = body length; SL = stylet length; V = Position of vulva; TL = tail length; DEGO = dorsal esophageal gland orifice; EP excretory pore; MW = maximum width; ESOP = Esophageal length; AW = Anal Width; *a* = body length/maximum body width; *b* = body length/esophageal length; *c* = body length/tail length *c*′ = tail length/anal body width

Figures in red indicate correlation values greater than 0.7 or less than −0.7

**Table 5:** Correlations between variables and components

	Female		Male	
	
	PC1	PC2	PC1	PC2
L	0.564	0.596	0.499	0.663
SL	0.562	0.105	0.675	0.087
V	0.714	0.318	0.463	−0.198
TL	0.482	−0.387	0.947	−0.079
DEGO	0.630	0.073	0.776	−0.046
EP	−0.326	0.369	−0.067	0.228
MW	0.614	−0.365	0.175	−0.563
ESOP	0.374	0.305	0.181	−0.116
AW	0.786	−0.064	0.682	−0.163
*a*	−0.161	0.773	0.245	0.899
*b*	−0.369	−0.070	0.171	0.556
*c*	−0.143	0.732	−0.873	0.287
*c*′	−0.729	−0.004	0.730	−0.010

L = body length; SL = stylet length; TL = tail length; DEGO = dorsal esophageal gland orifice; EP excretory pore; MW = maximum width; ESOP = Esophageal length; AW = Anal Width; *a* = body length/maximum body width; *b* = body length/esophageal length; *c* = body length/tail length *c*′ = tail length/anal body width

Figures in red indicate correlation values greater than 0.7 or less than −0.7

**Table 6:** One-Way ANOVA table for nine female and male populations from Alabama and one population from Mississippi (MSU)

POPULATION	L	SL	TL	DEGO	EP	MW	ESOP	AW	*a*	*b*	*c*	*c*′
BELLMINA	1.4	14.8*	47.8**	1.5	1.1	36.3**	3.9	9.5*	34.8**	4.3	41.9**	148.4**
SHAW	2.3	84.5**	0.7	7.9*	4.9*	13.4*	49.6**	2.3	12.0*	73.1**	3.5	91.6**
MURPHY	2.8	123.1**	17.0*	4.7*	0.6	16.5*	7.5*	0.4	2.3	1.7	6.9*	149.7**
HARGRAVE	1.6	184.4**	10.6*	20.4**	14.0*	12.7*	33.1**	1.0	13.9*	35.4**	10.9*	11.4*
THORTON	2.5	78.8*	12.4*	9.0*	13.1*	8.9*	45.9**	1.0	11.4*	36.7**	14.7*	6.7*
HAMILTON	2.6	74.5**	3.2	3.9	6.7*	4.4*	12.8*	0.0	11.2*	18.6*	6.7*	3.1
LAMONS	1.6	65.6**	0.5	0.1	0.5	7.7*	7.0*	1.8	8.1*	13.3*	1.5	114.6**
WHITEHEAD	4.2*	84.6**	1.3	8.6*	8.6*	7.3*	8.4*	1.0	11.8*	15.3*	4.1*	0.5
HUXFORD	0.0	0.2	80.3**	15.5*	0.8	8.1*	0.1	26.1**	4.6*	1262.4**	76.2**	224.3**
MSU	0.2	63.6**	49.9**	3.0	4.7*	11.1*	19.8**	18.0*	10.3*	40.3**	35.8**	189.6**

L = body length; SL = stylet length; TL = tail length; DEGO = dorsal esophageal gland orifice; EP excretory pore; MW = maximum width; ESOP = Esophageal length; AW = Anal Width; *a* = body length/maximum body width; *b* = body length/esophageal length; *c* = body length/tail length *c*′ = tail length/anal body width.

* Significant (0.05),

** Highly Significant (0.0001)

**Table 7:** Coefficient of variation for nine male and female populations from Alabama and one population from Mississippi (MSU)

POP	L	SL	TL	DEGO	EP	MW	ESOP	AW	*a*	*b*	*c*	*c*′
BELLEMINA	6.6	10.9	14.4	8.7	13.7	10.1	17.0	13.7	11.3	15.0	14.2	20.8
SHAW	6.6	8.9	12.2	10.7	8.8	6.8	8.2	13.2	9.9	7.9	11.6	17.4
MURPHY	7.8	8.3	9.5	12.6	11.4	6.6	8.6	13.9	9.1	8.6	9.9	13.6
HARGRAVE	6.5	6.3	8.5	7.4	6.2	5.7	8.7	5.2	7.3	9.4	10.7	9.3
THORTON	6.7	6.0	8.1	6.9	6.9	6.7	7.1	5.2	8.8	9.7	10.6	9.6
HAMILTON	7.4	7.4	8.4	9.2	6.4	9.1	8.0	0.0	9.4	9.4	10.9	8.4
LAMONS	7.3	8.3	13.2	10.2	8.0	7.3	12.6	14.3	10.4	11.7	13.6	19.2
WHITEHEAD	6.7	6.5	10.6	9.8	6.4	7.7	10.3	5.2	9.9	11.9	11.3	11.8
HUXFORD	8.1	20.2	15.2	14.0	14.4	12.0	13.3	17.9	14.2	10.6	16.0	19.0
MSU	6.8	11.4	16.6	13.5	9.8	7.9	28.8	17.1	8.9	17.3	19.7	20.5

L = body length; SL = stylet length; TL = tail length; DEGO = dorsal esophageal gland orifice; EP = excretory pore; MW = maximum width; ESOP = Esophageal length; AW = Anal Width; *a* = body length/maximum body width; *b* = body length/esophageal length; *c* = body length/tail length *c*′ = tail length/anal body width,

**Table 8:** Mean and standard deviation values of nine male and female populations from Alabama and one population from Mississippi (MSU)

Population (n=20)	L (μm)		SL (μm)		TL (μm)		DEGO (μm)		EP (μm)		MW (μm)		ESOP (μm)		AW (μm)	
							
	Mean	S.D.	Mean	S.D.	Mean	S.D.	Mean	S.D.	Mean	S.D.	Mean	S.D.	Mean	S.D.	Mean	S.D.
BelleMina (F)	381.0^ab^†	26.1	16.3b^cd^	1.8	33.4^a^	4.7	34.4^bcd^	2.4	66.4^bc^	8.1	16.9^a^	2.0	124.5^b^	26.3	11.4^a^	1.5
BelleMina (M)	390.5^a^	24.8	14.3^ab^	1.5	45.9^a^	6.6	35.6^ab^	3.6	63.4^b^	9.6	13.9^bc^	0.9	112.0^ab^	11.1	9.9^a^	1.5
Shaw (F)	375.5^abc^	22.6	17.6^ab^	1.6	30.8^ab^	3.1	36.7^ab^	4.3	69.1^ab^	5.1	15.8^abc^	1.2	125.0^b^	10.0	9.6^b^	1.2
Shaw (M)	387.5^a^	27.3	13.6^abc^	1.1	29.9^b^	4.2	33.3^bc^	3.1	65.0^ab^	6.7	14.7^ab^	0.9	104.0^b^	8.8	9.0^ab^	1.3
Murphy (F)	381.5^ab^	30.3	16.9^bc^	1.1	29.6^abc^	2.8	36.1^bc^	5.2	68.8^ab^	9.7	16.2^abc^	1.0	126.0^b^	10.5	8.7^bc^	1.2
Murphy (M)	366.0^a^	28.2	12.6^cd^	1.4	26.2^bc^	2.4	33.1^bc^	3.3	70.8^a^	5.8	14.9^ab^	1.1	117.0^a^	10.3	8.4^bc^	1.2
Hargrave (F)	364.0^abc^	26.8	15.6^cde^	1.2	28.4^bc^	2.5	30.8^de^	2.5	73.4^a^	4.2	15.2^bcd^	1.2	123.0^b^	12.6	7.1^d^	0.0
Hargrave(M)	373.5^a^	20.3	11.9^d^	0.0	26.1^bc^	2.1	27.7^d^	1.8	68.2^ab^	4.7	14.3^abc^	0.0	105.0^b^	6.1	7.3^d^	0.5
Thorton (F)	356.0^bc^	23.7	14.3^e^	0.0	29.0^bc^	1.7	30.1^e^	2.5	73.7^a^	5.3	15.5^abcd^	1.2	123.5^b^	9.3	7.3^d^	0.5
Thorton (M)	368.0^a^	24.6	11.9^cd^	0.0	26.5^bc^	2.7	28.2^d^	1.4	68.1^ab^	4.5	14.5^ab^	0.7	106.0^b^	6.8	7.1^d^	0.0
Hamilton (F)	361.0^abc^	26.5	15.0^de^	1.1	28.3^bc^	2.4	32.1^de^	2.4	72.0^ab^	4.1	14.3^d^	1.3	121.0^b^	10.2	7.1^d^	0.0
Hamilton (M)	375.0^a^	28.2	12.3^cd^	0.9	27.0^bc^	2.2	30.3^cd^	3.3	68.3^ab^	4.9	13.4^c^	1.2	110.5^ab^	8.3	7.1^d^	0.0
Lamons (F)	359.5^abc^	25.6	16.7^bcd^	1.1	29.3^bc^	4.0	33.3^bcde^	2.9	71.0^ab^	6.3	15.4b^cd^	1.2	125.0^b^	17.0	8.2^cd^	1.2
Lamons (M)	370.0^a^	27.5	13.4^bc^	1.4	28.4^bc^	3.6	32.9^bc^	3.8	69.7^ab^	4.9	14.4a^bc^	0.9	112.5^ab^	12.5	7.7^cd^	1.1
WhiteHead (F)	349.5^c^	26.3	14.4^e^	1.2	25.9^c^	3.3	31.4^de^	3.3	72.1^ab^	4.8	14.9^cd^	1.3	116.0^b^	10.0	7.3^d^	0.5
WhiteHead (M)	365.0^a^	21.6	11.9^d^	0.0	25.0^c^	2.0	28.7^d^	2.1	67.9^ab^	4.1	13.9^bc^	0.9	105.5^b^	12.8	7.1^d^	0.0
Huxford (F)	384.0^a^	29.8	15.4^cde^	3.4	31.1^ab^	5.3	32.4^cde^	4.0	70.8^ab^	9.9	16.3^abc^	2.2	107.9^b^	18.8	12.7^a^	2.5
Huxford (M)	384.5^a^	32.7	14.9^a^	2.7	48.2^a^	6.6	38.6^a^	5.8	67.9^ab^	10.0	14.6^ab^	1.4	109.0^ab^	7.9	9.5^a^	1.3
MSU (F)	386.5^a^	28.2	18.9^a^	2.0	33.4^a^	5.9	39.9^a^	5.5	59.9^c^	6.6	16.4^ab^	1.3	166.0^a^	55.3	11.5^a^	2.2
MSU (M)	390.0^a^	24.7	14.2^ab^	1.8	48.7^a^	7.7	37.0^a^	4.9	64.1^b^	5.6	15.1^a^	1.2	110.0^ab^	10.3	9.2^ab^	1.2

L = body length; SL = stylet length; TL = tail length; DEGO = dorsal esophageal gland orifice; EP = excretory pore; MW = maximum width; ESOP = Esophageal length; AW = Anal Width.

† Means followed by a different letter are significantly different at the 5% level using the Tukey’s Studentized Range (HSD) Test
